# Advanced Fractionation of Kraft Lignin by Aqueous Hydrotropic Solutions

**DOI:** 10.3390/molecules28020687

**Published:** 2023-01-10

**Authors:** Rita Gaspar, Marcelo Coelho dos Santos Muguet, Pedro Fardim

**Affiliations:** 1 Chemical and Biochemical Reactor Engineering and Safety (CREaS), Department of Chemical Engineering, KU Leuven, Celestijnenlaan 200f, P.O. Box 2424, 3001 Leuven, Belgium; 2 Klabin S.A., Paraná 84275-000, Brazil

**Keywords:** hydrotropic fractionation, Lignoboost Kraft lignin, sodium xylene sulfonate, sodium cumene sulfonate, molecular weight, GPC, NMR

## Abstract

Lignin is an underutilized high-potential biopolymer that has been extensively studied over the past few decades. However, lignin still has drawbacks when compared with well-known petroleum-based equivalents, and the production of tailored lignin fractions is highly in demand. In this work, a new method for the fractionation of Lignoboost Kraft Lignin (LKL) is proposed by using two different hydrotropes: sodium xylenesulfonate (SXS) and sodium cumenesulfonate (SCS). The different fractions are obtained by sequentially decreasing the hydrotropic concentration with the addition of water. Four and three different fractions were retrieved from the use of SXS and SCS, respectively. The LKL and respective fractions were analysed, and compared by GPC, FTIR-ATR, ^1^H-NMR, ^13^C-NMR, ^31^P NMR, 2D HSQC and SEM. The fractions showed different molecular weights, polydispersity, and amount of functional groups. Our water-based lignin fractionation platform can potentially be combined with different lignin extraction and processing technologies, with the advantage of hydrotrope recycling.

## 1. Introduction

Lignin is, together with cellulose and hemicelluloses, one of the three main components of plant cell walls. Its main functions within plants are to provide cell wall rigidity, assist with the transportation of minerals through the plant vascular system, and protect the plants against stress, as well as virus and pathogens [1]. As a biopolymer with anti-UV, antimicrobial, and antioxidant properties, lignin has the potential to be used in a wide range of applications throughout different industries, from chemical conversion to biomedical applications [2]. The interesting properties and the high availability of lignin (primarily as a byproduct of the paper and pulp industries) make this biopolymer one of the most underestimated raw materials nowadays.

While the world is slowly shifting to the use of non-petroleum-based products, lignin heterogeneity and variability within different species of plants are the main drawbacks when switching to the use of this biopolymer as a raw material. Lignin is mainly comprised of three different phenylpropane units: guaiacyl, syringyl, and p-hydroxyphenyl units bonded through different interunit linkages units (β-O-4′, β-β′,β-5′, etc.) [3]. The exact percentages of these monomeric units and interunit linkages vary within softwood, hardwood, and with the lignin extraction process (e.g., Kraft, Sulfite, or Organosolv).

Despite the high interest in lignin research and lignin availability, and consequent low cost, its actual use on an industrial scale is still very limited. Of the nearly 100 million tons/year of Kraft lignin produced worldwide, approximately 98% is burned as fuel [2]. The remaining low percentage of lignin is used as an additive for adhesives and cements, food and feed, and dispersants [4]. 

Lignin is separated from cellulose and hemicellulose using sodium hydroxide and sodium sulfide in the Kraft process and the bottleneck of the operation is the surplus of black liquor in the recovery boiler. Thus, the extraction of lignin from black liquor to reduce the calorific value and solids load in the recovery boiler is a solution to increase the pulping capacity [5]. The possible revenues from the use of purified lignin in high-added-value products and applications are the driving forces for the development of processes to isolate lignin from black liquor. Nowadays, the three main extraction processes of lignin from black liquor are the WestVaco process [6], the LignoBoost process [7], and the LignoForce SystemTM [8]. Nevertheless, the high molecular weight (Mw) and polydispersity of these lignins are still drawbacks when using them in potential applications in which Mw-dependent polymer properties are important. However, developing separation processes that can fractionate kraft lignin into selective fractions with specific Mw and properties has recently gained interest from researchers. 

Most processes for the fractionation of Kraft lignin are based on the solubility of the different lignin fractions in several organic solvents [9,10,11,12] or in membrane separation processes from black liquor [13,14,15,16,17]. While the use of organic solvents produces well-defined fractions of lignin, the safety and handling of this type of process at an industrial scale is a disadvantage. Regarding the membrane processes, the fouling and, consequently, the cost of constantly cleaning the membranes can lead to higher maintenance costs. 

Hydrotropes are organic salt compounds that increase the solubility of otherwise insoluble components in water [18]. They are biodegradable, reusable non-toxic chemicals that can be recovered and recycled a high number of times. Interestingly, by lowering the hydrotrope concentration it is possible to retrieve the dissolved solute without losing its hydrotropic power [19]. For those reasons, recently, different research groups have been developing lignin extraction from biomass using different hydrotropes, such as sodium xylene sulfonate and p-toluenesulfonic acid. While different extraction conditions originated different lignin yields, they also created lignin with different properties, such as molecular weight and chemical composition [20,21,22]. However, to the best of our knowledge, the solubilization of Lignoboost Kraft Lignin (LKL) and subsequent fractionation of LKL using hydrotropes has not been reported yet. Based on the premise that lowering the hydrotropic concentration by dilution with water would produce lignin with specific characteristics, a method was developed and reported for the first time in this paper for fractionating LKL by performing sequential dilutions of hydrotropic concentration with water.

## 2. Results and Discussion

### 2.1. Hydrotropic Fractionation

LKL was initially solubilized in hydrotropes, and the rational for this fractionation involved the identification of the precise amounts of water that needed to be added to the solution to cause the precipitation of LKL fractions with different Mw. The concentration of lignin in the initial solution was chosen by finding the solubility limit of lignin in the hydrotropic solutions. Above the hydrotropic concentration of 40 wt%, the maximum amount of solubilized lignin did not change significantly, and for that reason the initial hydrotropic concentration was kept at 30 wt%. For the hydrotropic fractionation of LKL using sodium xylene sulfonate, the 30 wt% hydrotropic solutions with a concentration of lignin of 150 g/L were first diluted to 20 wt%. However, since no lignin fraction precipitated, this first dilution step was performed sequentially in 2 wt% decremental steps until a fraction was precipitated. The first fraction to precipitate (with the highest yield) was at 16 wt% of SXS. This fraction was separated by centrifugation and the supernatant collected. The supernatant, with a concentration of 16 wt% of SXS, was then diluted to 14 wt% SXS, and the dilution was based on the amount of hydrotrope and water and not considering the lignin in solution. The 16 wt% fraction (precipitate) was washed with an excess of deionized water to remove the remaining hydrotrope, and freeze-dried for further characterization. The amount of water required to remove all the hydrotrope was confirmed by analysing the cleaning fractions using FTIR-ATR and later ^1^H-NMR. The fraction was considered clean when no peaks associated with the hydrotrope were visible in the FTIR and NMR spectra. The main advantage of this washing step is that, depending on the application in which the lignin will be used, the fraction can be cleaned to a specific degree of purity.

The same procedure of fractionation and purification of the obtained fractions was applied for further dilutions, and four different fractions were obtained with hydrotropic concentrations of 16, 14, 12 and 10 wt% of SXS. No fraction was retrieved for 8 wt%, and for that reason the remaining supernatant retrieved after the 10 wt% dilution was not used for any further dilution steps and characterization. 

The method used for the fractionation of LKL using sodium cumene sulfonate was the same as for the fractionation using SXS. However, the first fraction that was possible to retrieve after sequential dilution steps from 30 wt% SCS solution with 150 g/L of LKL was at a hydrotropic concentration of 10 wt%. The following retrieved fractions were at 8 and 6 wt%, meaning that only three fractions were retrieved from the fractionation using this hydrotrope. No fraction was retrieved from diluting the 6 wt% to 4 wt%, and so the remaining supernatant was not recovered. 

The following table (Table 1) shows the obtained yields for the retrieved fractions for both hydrotropic fractionations using SXS and SCS. The fraction yield (%) was calculated based on the amount of collected clean fraction after freeze-drying, and compared with the initial amount of lignin added to the solution. 

It could be argued that the chemical structure of the two hydrotropes [23,24], which differ only on the alkyl chain attached to the benzene ring, is the main cause for the different degree of solubilization of this type of Kraft lignin and, thus, results in lignin fractions precipitating at different hydrotropic concentrations for both hydrotropes. However, the exact way in which the hydrotropy mechanism of these two different hydrotropes works still needs to be investigated as different reasons might explain this solubility-based fractionation process. 

First, the minimum hydrotropic concentration (MHC) is hydrotrope-dependent. This means that a hydrotrope with a similar chemical structure but a different number of alkyl chains can have a different MHC. Based on the literature, the MHC is lower for hydrotropes with longer alkyl chains [23]. In this particular study, xylene sulfonate is an alkylbenzene sulfonate with two methyl groups bonded to two different carbons from the aromatic ring (C_8_H_9_), while cumene sulfonate has an isopropyl group attached to the benzene ring (C_9_H_11_). This would indicate that a lower concentration of SCS (longer alkyl chain) would be needed to solubilize higher molecular weight lignins, explaining why the first lignin fractions that precipitate with SCS are at a lower hydrotropic concentration than with SXS. 

However, to better understand the effect of the alkyl chain length on the fractionation yield, more alkylbenzene sulfonates with longer alkyl chains should be used for the fractionation of kraft lignin. Additionally, the maximum concentration of a solute in a certain hydrotropic solution also varies within different solutes [25]. It could be considered that there is an equilibrium between the concentration of the hydrotrope and the concentration of each lignin in solution, that is disrupted for specific hydrotropic concentrations. 

A study on the efficiency of different hydrotropes (particularly SXS and SCS) on the delignification of cotton stalks [26] concluded that using SCS in the delignification process produced a higher reducing sugars yield, meaning that less lignin was available in the system for the enzymes used in the enzymatic hydrolysis to bind to. In the same work, it was also concluded that an equilibrium between the maximum amount of initial biomass and the extraction efficiency of lignin was needed to optimize the process, which is in line with the hypothesis that the solubility of lignin in the two hydrotropes is based on an equilibrium of the system water/hydrotrope/lignin. 

The availability of lignin hydroxyl groups to interact with the hydrotropic solution can also play a role in the fractionation process. Research by Petridis et al., applying molecular dynamics simulations, showed that lignin with a higher degree of polymerization displays a spherical conformation, while lignin with shorter chains presents an aspherical conformation [27]. This could mean that lignin with higher molecular weight will have fewer available hydroxyl groups in this conformation to interact with the hydrotrope and, thus, could lead to precipitation. Another study that could confirm this hypothesis, published in 2020, demonstrates that the mechanism behind hydrotropy might be the aggregation of hydrotrope molecules around the solute, facilitated by water [28]. This would mean that, for the spherical long-chain lignin fractions, fewer hydrotrope molecules would be available in their immediate surroundings to aggregate around them, making them precipitate. 

Finally, another parameter that can be studied in the future is the effect of temperature increase on the obtained fractions with both hydrotropes. The effect of higher temperatures on delignification processes is well documented in the literature, with lignin extracted at higher temperatures having lower molecular weight [20,26] An increase in temperature, above 100 °C, could then break lignin into smaller Mw fractions, which could later be separated by our simple hydrotropic fractionation method. Studies on the effect of temperature on the aggregation behaviour of SCS [29] also show how the hydrotrope solubility itself can be completely modified by working at higher temperatures, which could lead to a whole new research study for this fractionation process. 

### 2.2. Characterization of LKL Fractions

After washing and drying all the fractions, they were further characterized using different advanced analytical techniques.

#### 2.2.1. GPC

The molecular weight and polydispersity were analysed by SEC-GPC. Based on the principle that the solubility of a polymer in certain solvents depends on the molecular weight of the polymer, the different fractions of lignin precipitate when the concentration of hydrotrope in solution is no longer sufficient to dissolve a specific molecular weight fraction. However, this hypothesis supposes that the different fractions already exist in the original LKL and are just separated based on solubility and selective precipitation. 

**Figure 1:**
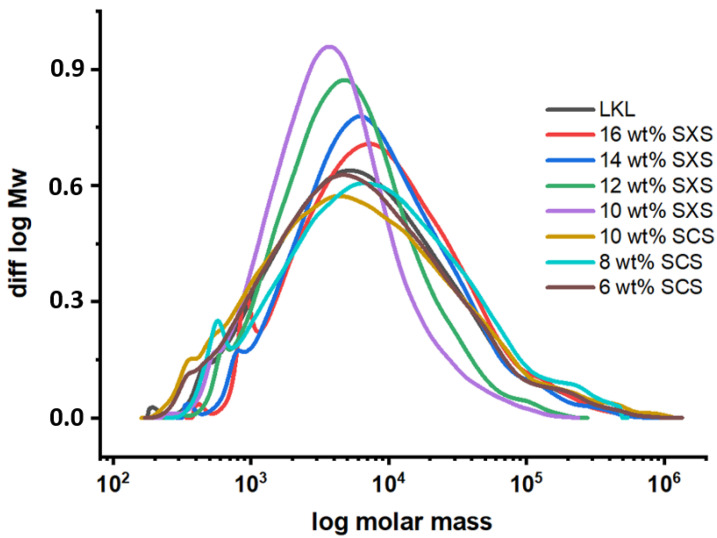
SEC-GPC results for LKL and fractions obtained from the hydrotropic fractionation using SXS and SCS.

As can be seen in Figure 1 and Table 2, the fractions obtained from lower hydrotropic concentrations have a lower molecular weight and polydispersity, with the fraction with the highest yield (16 wt% fraction) having a similar Mw to the original LKL but the fraction of 10 wt % having a Mw almost 3.5 times lower than the original fraction. This proves that the fractionation process by sequential dilution of hydrotropic concentration using water is possible. For the fractions obtained from the SCS fractionation method, the GPC results (Figure 1 and Table 2) show that the Mw does not decrease substantially when compared to the initial LKL; however, there is a significant decrease in the polydispersity. This could indicate that, in a hydrotropic solution of SCS, the lower molecular weight fractions are still dissolved in the final supernatant, which suggests that this specific lignin is more soluble in SCS than in SXS. It could also be that some of these lower molecular weight fractions are being dragged with the higher molecular weight fractions. In future research, further hydrotropic fractionation cycles on the precipitated fraction can be performed to understand if any residual low molecular weight fractions are being dragged in the precipitation of higher Mw fractions. 

It is important to mention that the challenges of the characterization of lignin Mw by GPC are well discussed in the literature [30]. First, the standards used for GPC analysis of lignin (commonly polystyrene) have a completely different molecular structure than lignin, producing over- or underestimated values of Mw and polydispersity. Additionally, the autofluorescence characteristic of lignin, in particular Kraft lignin, is known to adulterate the Mw results. For example, if the GPC equipment is equipped with a UV detector without fluorescence filters, the Mw of lignin samples can be overestimated. The type of solvent (mixture of solvents) used to dissolve the lignins will also play a role in the characterization of the lignins’ molecular weight. 

For this reason, the molecular weight of this LKL sample and consequent fractions cannot be considered an absolute and exact measurement. However, the values of the Mw and polydispersity of the lignin fractions can still be used for comparison. While the polydispersity value of the initial LKL is around 10, the one obtained PDI value for the fractions with the lowest Mw (12 and 10 wt% SXS) is about three times lower, meaning a narrower Mw distribution. Additionally, the Mw varies from an initial 24,000 g/mol to close to 7000 g/mol, proving that this type of fractionation process is possible. 

#### 2.2.2. Elemental Analysis

To further characterize the lignin fractions, an elemental analysis was performed. This analysis was performed in triplicate, and the results for the average percentage of each element and its standard deviation for the initial LKL and the fractions obtained using SXS and SCS are presented in Table 3. While the percentage of sulphur, nitrogen and hydrogen is practically constant, the percentage of carbon increases with the decrease of the molecular weight, which was not expected. One possible explanation for this is that some residual hydrotrope was still present in the dried and cleaned samples. While the hydrotrope removal was confirmed by ^1^H-NMR, it is possible that very low concentrations of hydrotrope were still present in the samples, despite the lack of peaks related to these compounds being present in the spectra. However, the standard deviation for the measurement of the percentage of carbon is also higher than for all the other elements, indicating that this difference might be related only to the deviation of the method itself and not to the different lignin fractions.

#### 2.2.3. NMR Characterization

Different NMR techniques were applied for the characterization of the different lignin fractions. Alongside the use of ^1^H NMR to evaluate the residues of hydrotrope and the efficiency of the cleaning cycle for hydrotrope removal in the obtained fractions, ^13^C NMR, ^31^P NMR, and 2D HSQC techniques were performed. 

The use of ^13^C NMR with specific sample conditions and the application of certain parameters can be used to identify and quantify several different lignin moieties [31], so this technique was first applied to the lignin fractions to verify whether there were any significant structural differences between the obtained fractions. The calculation of the different lignin moieties’ quantities was performed based on the calculations of the same article, which was performed in the following way: the aromatic region (100–163 ppm) in the ^13^C NMR spectra was integrated and the integral calibrated to a value of 600 (100 Ar unis contain 600 carbons). The regions of interest in the spectra are then integrated and this value is in the units of “per 100 Ar”. 

While the quantification of the lignin moieties should be calculated (for most moieties) as the difference between the amount calculated for the acetylated sample and the non-acetylated sample [31], it was noted that there could be a small interference from residual hydrotrope in the spectra of the non-acetylated samples. The sensitivity of this particular ^13^C NMR analysis, with an extremely high number of scans and lignin concentration, is higher than the one performed for the ^1^H NMR washing experiments. Then, it is possible that the ^13^C NMR spectra of non-acetylated samples will contain peaks that would otherwise not be present in the ^1^H NMR spectra. This would be shown as small, almost imperceptible, peaks in the aromatic region of the spectra (100–163 ppm) that would nevertheless interfere with the calculation of the different moieties. 

However, for the acetylated samples, as the solvent system used for the acetylation is pyridine/acetic anhydride, and the acetylation is followed by extensive washing with ethanol, it is ensured that any vestigial hydrotrope is completely removed from the sample and, thus, will not appear in the ^13^C NMR spectra. For this reason, the moieties that were calculated through the ^13^C NMR spectra were calculated only using the acetylated samples. 

**Figure 2:**
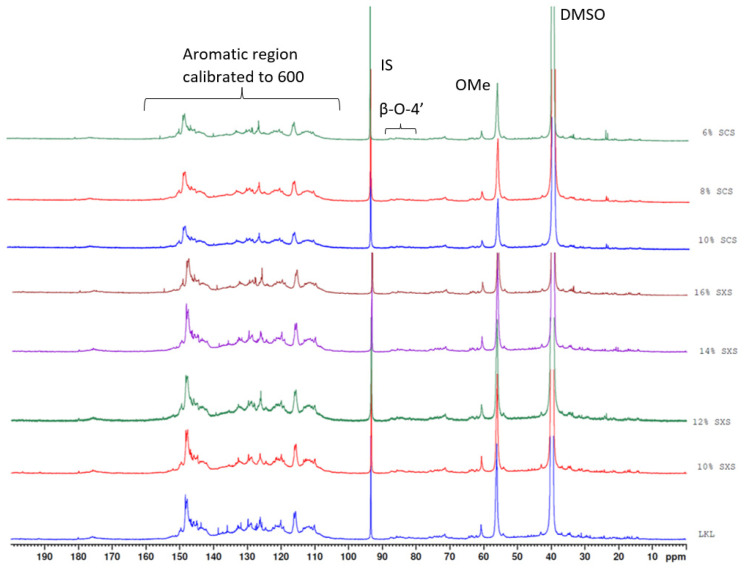
C NMR spectra of fractions from SXS and SCS hydrotropic fractionation.

In Figure 2, the spectra of the different obtained fractions of lignin samples are shown. The main goal of this characterization method when researching lignin fractionation is to evaluate the quantities of Aliphatic OH, Phenolic OH and Carboxyl groups, which are associated with lignin reactivity. Based on the literature [32], it is expected that lignin fractions with lower Mw have higher content in Phenolic OH and Carboxyl groups and lower content in Aliphatic OH. However, since there is no significant difference between the spectra of the fractions and the spectrum of the initial LKL, the quantification of these functional groups using ^13^C NMR did not provide precise results, and so the quantification of these functional groups was made using ^31^P NMR technique. Since the ^31^P NMR technique provides simpler spectra, in which only the hydroxyl groups are shown, it reduces the possible errors of quantification of functional groups in the ^13^C NMR spectra of the different Kraft lignin samples with such similar spectra. 

**Figure 3:**
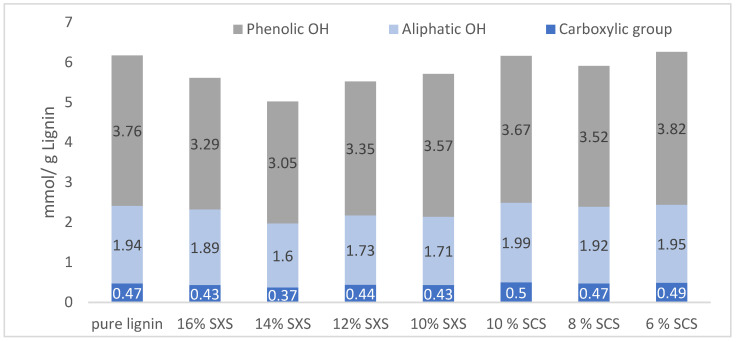
Quantification of phenolic, aliphatic, and carboxylic OH groups in LKL, SXS and SCS fractions.

As the quantification of different lignin moieties using ^13^C NMR is a very complex technique, because of the overlapping groups in the NMR spectrum and because the difference between the quantity of functional groups is very similar for these fractions, the groups responsible for lignin reactivity were quantified using ^31^P NMR, following the protocol from the literature [33]. In Figure 3, the amount of the phenolic, aliphatic and carboxylic OH groups are shown for all the fractions in mmol/g lignin. 

The quantification of interunit linkages was still performed using the ^13^C NMR spectra of the acetylated samples. The three main interunit linkages in lignin are β-O-4′, β-5 (phenylcoumarane) and β-β (resinol). Based on the literature [31,34], the resinol and phenylcoumarane units can be calculated based only on the spectra of the acetylated samples, while β-O-4′ should be calculated as the difference between both acetylated and non-acetylated samples. The results of the quantification of the interunit linkages of the different lignins can be seen in Table 4. Based on these results, there is only a significant reduction in the amount of interunit linkages for the samples with the lowest molecular weight (12 and 10 wt% SXS), while no significant difference can be found in the samples with higher Mw. It is important to mention that the quantification of the β-O-4′ was made only using the acetylated samples, which means that these values should be slightly lower (if the amount of β-O-4′ in non-acetylated samples was also considered). However, the difference should not be significant. The spectra of all the fractions obtained with ^13^C NMR can be found in the Supplementary Information (Figures S1–S4) as well as the spectra of the pure hydrotropes (Figures S14 and S15).

Regarding the results about the ^31^P NMR (Figure 3), based on the literature for other lignin fractionation processes, it was expected that the lower the molecular weight of the obtained fraction, the lower the aliphatic content. In the opposite trend, the lower the molecular weight, the higher the expected phenolic content [12]. The group quantification of the different fractions obtained by hydrotropic fractionation shows a similar trend. The fractions obtained by hydrotropic fractionation with SXS show a lower content of aliphatic OH for the fractions with lower Mw (12 and 10 wt%), and a higher quantity of phenolic groups for these two fractions. For the fractions obtained by fractionation with SCS, the samples have approximately the same amount aliphatic OH groups, while there seems to be a slight increase in the amount of phenolic OH for the fraction of 6 wt% SCS. The results for the fraction of 14 wt% do not follow a similar trend to all the other fractions, however, and this could indicate an error during the preparation of the sample or with the sample itself, and not exactly a difference in the fraction. ^31^P NMR, while a high-sensitivity technique, also has some parameters that can affect the stability of the sample. For once, the sample is not stable for longer periods of time after being prepared in the NMR tube [33]. Additionally, while all the samples were freeze-dried for several days, it can be argued that that specific 14 wt% fraction could still contain some moisture, which would interfere with the ^31^P NMR results. The spectra of all the fractions obtained with ^31^P NMR can be found in the Supplementary Information (Figures S5 and S6).

The carboxylic group quantification does not show a significant variation between fractions. As is known from the literature [35], it is expected that the fractionation processes of Kraft lignin will have a higher influence on the quantification of functional groups. However, this fractionation process is a mild process based on the solubility of lignin in the hydrotropes, so no significant differences in the number of functional groups were expected. As with the fractionation of lignin with organic solvents [11,36,37], the fractionation itself is based on the solubility of higher and lower Mw fractions on the organic solvents and, so, the exact fractions with specific Mw are already present in the initial lignin. In this fractionation process, a similar conclusion can be made, as no physical modifications are present in the lignins after the fractionation. While this can be a disadvantage for the possible fractions that can be obtained, it opens the doors to exploring how different technical lignins and different hydrotropes would allow the fractionation of lignin fractions with tailored Mw. 

**Figure 4:**
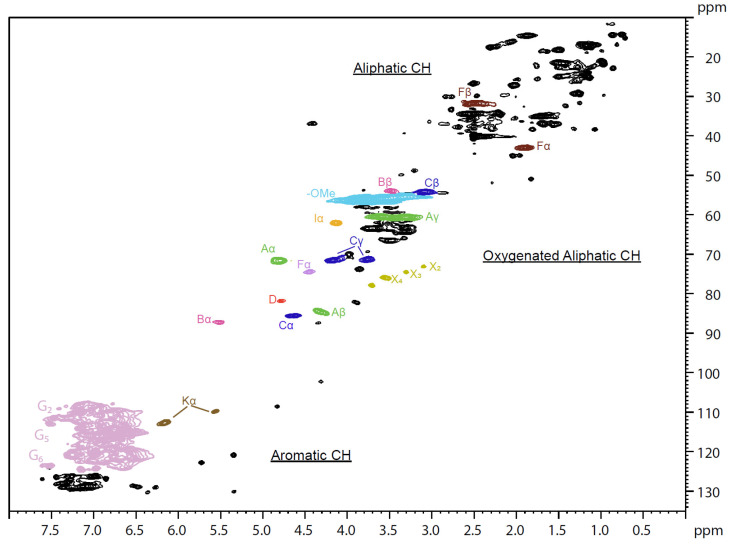
The 2D HSQC of initial Kraft lignin, before fractionation.

**Figure 5:**
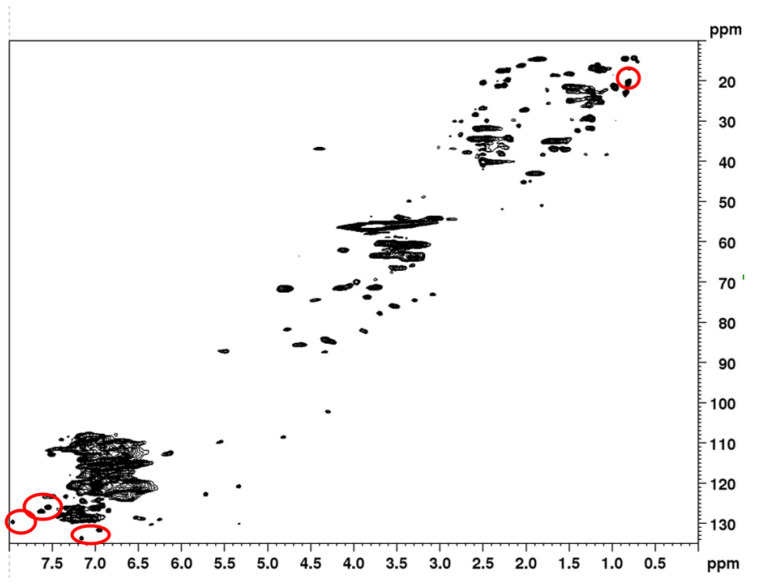
The 2D HSQC of 10% SXS fraction.

Additionally, a 2D HSQC was performed as a method to understand if there were any chemical structural modifications to the fractions that were not visible in the other characterization methods. In Figure 4, the 2D HSQC spectrum of the original LKL is presented. In Figure 5, the 2D HSQC spectrum of the 10% SXS fraction obtained from the fractionation using SXS is presented, just to provide an overall visual comparison between the initial lignin and a fraction. The remaining spectra for the 2D HSQC can be found in the Supplementary Information (Figures S7–S13). As expected, no significant differences between the spectra were found. By analysing the intensity of the peaks related with the β-O-4′, β-5′ and β-β substructures, the intensity of the peaks is lower in the fractions with lower molecular weights, which was also expected, and is in accordance with the results from the ^13^C NMR. The C-H bonds related to the β-O-4′ substructures are identified from Aα to Aγ. One of the other most common chemical bonds in the lignin structure, β-5′ substructures, are identified as Bα and Bβ in the zones of the spectra 86.5/5.52 and 53.3/3.48, respectively. For the ranges 33.8–42.2/1.91–2.54, the C-H bonds related to the secoisolariciresinol structure are identified as Fβ and Fα, respectively. The area of the 2D HSQC spectra in which three different structures are identified with the letter X is related to Kraft lignin carbohydrates (xylosyl units). The identification of the lignin substructures in the 2D HSQC spectra was made based on [12].

As mentioned in the ^13^C NMR results, it is still possible to see some vestigial hydrotrope peaks in the 2D HSQC spectra (marked as red circles in Figure 5), which proves that the quantification using the ^13^C NMR of non-acetylated lignin samples could have some interference from the residual hydrotrope in the sample. The exact areas of the 2D HSQC spectra related to the hydrotropes could be identified by comparing the ^13^C NMR and ^1^H NMR of both pure hydrotropes with the corresponding positions in the 2D HSQC spectra. These regions are clearly indicated in Figure 5, by a circle around these peaks. The region of these peaks for the other fractions and samples with SCS can be found in the Supplementary Information (Figures S7–S13), as well as the spectra of the pure hydrotropes. 

The only significant difference between the original lignin and the spectra of the fractions is that, in the region denoted with an X (corresponding to carbohydrates), the peaks are not present in the fraction of 10 wt% SCS (higher Mw) and are present in lower intensities in the spectra of the other two SCS fractions. This could indicate that the remaining carbohydrates are mostly still dissolved in the final SCS supernatant. 

Samples of SCS have a lower intensity of the carbohydrate content, with the 10 wt% SCS showing no peaks at all compared with this in the oxygenated aliphatic area. This trend is not visible in the fractions obtained from SXS. 

In the Aliphatic CH area, once again the only difference between the spectra that can be seen between the original lignin and the fractions is related to the hydrotropes. 

**Figure 6:**
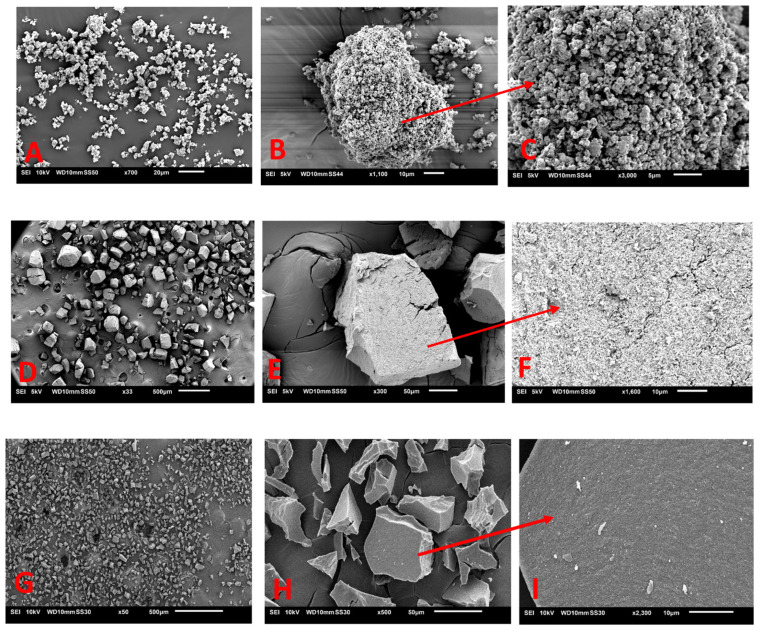
SEM images of LKL and lignin fractions. (**A**–**C**) Initial Lignoboost Kraft lignin. (**D**–**F**) Fraction of 14% SXS. (**G**–**I**) Fraction of 8% SCS.

**SEM.** For the SEM images, only the original lignin and one fraction from each hydrotropic fractionation are presented (14% SXS and 8% SCS) in Figure 6A–I, since all other fractions gave SEM similar images. As can be seen in Figure 6, there is a slight difference between the original lignin and the fractions in relation to the shape of the particle. However, this is only a cause of the drying method, with LKL being obtained by spray drying in a Lignoboost pilot plant while the obtained fractions were freeze-dried in a laboratory sized freeze dryer. The freeze-drying method provides particles with sharp edges, although it is possible to see in Figure 6F,I that the bigger particles are an agglomerate of smaller particles in the nm range. Regarding the differences between the types of dried particle obtained from fractionation with both hydrotropes, the particles obtained from the SCS fractions seem to have a more compact structure when compared with the SXS fraction. Additionally, the samples from SCS fractions appear to have a more defined particle size distribution than the particles observed for SXS. It is possible that the fractions’ particles are agglomerates of well-defined nanoparticles; however, this was not tested. 

## 3. Materials and Methods

### 3.1. Materials

Softwood Kraft lignin obtained from the Lignoboost process was used. LKL was dried using spray drying and stored at 4 °C. Sodium xylene sulfonate was purchased from Sigma-Aldrich, and sodium cumenosulfonate (40 wt% solution) was kindly supplied by HOESCH (Düren, Germany). All the chemicals used in NMR and GPC analysis are presented in the Section 3.2. 

### 3.2. Methods

#### 3.2.1. Hydrotropic Fractionation

A solution of 30 wt% in water of either SXS or SCS was prepared at room temperature. This concentration was chosen based on the solubility limit of the hydrotropes in water. Lignin was added to the hydrotropic solution at a concentration of up to 150 g/L, and the solution was heated and stirred at 50 °C for 10 min. 

A sequential fractionation process was used, in which, after diluting to each specific concentration, the soluble and insoluble fractions were separated by centrifugation at 6000 rcf. After centrifugation, the supernatant was collected and used for the following dilutions. Simply, a 30 wt% hydrotropic solution of SXS with a concentration of 150 g/L of lignin was diluted with water in a stepwise decrease of 2 wt%. Once the first lignin fraction precipitated, the following fractions were retrieved by continuing the stepwise decrease process. The samples were then centrifuged at 6000 rcf for 100 min and, after centrifugation, the precipitate was further washed with water to remove the remaining hydrotrope and freeze-dried. The supernatant was then diluted to 2 wt% lower than the previous concentration. For the fractionation with SXS, that entails the dilution of the supernatant from 16 wt% to 14 wt% of SXS. After dilution, the same procedure of centrifugation, separation, and cleaning of the fraction was applied. Four different fractions were obtained by sequentially decreasing the hydrotropic concentration: 16, 14, 12 and 10 wt %. The remaining supernatant was not further analysed. 

**Figure 7:**
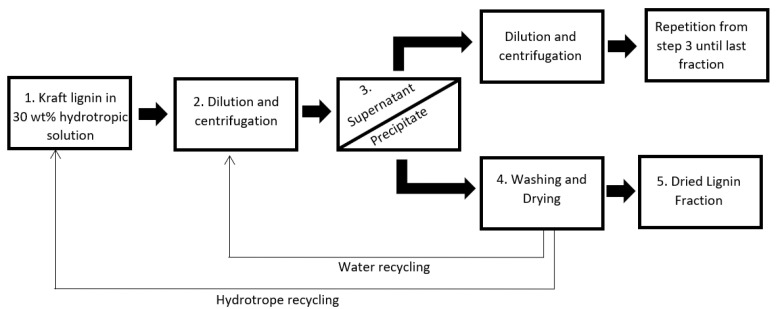
Diagram of hydrotropic fractionation process.

The same experimental procedure was applied to the 30 wt% hydrotropic solution of SCS, but the concentrations of the hydrotrope in the different fractions were 10, 8 and 6 wt%. All the different fractionation steps were performed at room temperature. Figure 7 shows a diagram of the fractionation process. 

#### 3.2.2. Fractions Characterization

The lignin fractions and original Lignoboost Kraft Lignin (LKL) were characterized using different techniques, such as FTIR-ATR, NMR, SEM, GPC, and elemental analysis. 

**FTIR-ATR.** To understand the efficiency of the hydrotrope removal from the washing step, the 8 lignin samples and pure hydrotropes were analysed by FTIR-ATR to compare their chemical structures. The equipment used was a Bruker Alpha. The number of scans was 24, with a resolution of 4 cm^−1^. 

**NMR.** ^1^H-NMR and quantitative ^13^C-NMR were performed for all the fractions retrieved from SXS and SCS and for the original lignin sample. Regarding the ^1^H-NMR, analysis spectra were recorded at 25°C in a Bruker Avance III HD 400 by dissolving around 80 mg of lignin in 0.55 mL of deuterated DMSO.

For ^13^C-NMR analysis, the samples were prepared as in the procedure found in the literature [31]. For all the different lignins (8 in total), two types of samples were prepared: acetylated and non-acetylated lignin. Essentially, for the acetylation of the lignin samples, a mixture of 1:1 (*v*/*v*%) of anhydrous pyridine/acetic anhydride, with a total of 4 mL of solution, was mixed with 150–200 mg of dried lignin. The solution was stirred for 24 h at room temperature. After, ethanol was used to remove the pyridine and any traces of acetic anhydride. A total of 10 mL of ethanol were added, and the solution was stirred for an additional 30 min. The ethanol was evaporated, and this procedure was repeated between 7 and 8 times until the lignin was completely clean and dry. Finally, the samples were washed with water and freeze-dried. 

For the ^13^C-NMR procedure, 200 mg of acetylated or non-acetylated lignin were mixed with 0.50 mL of deuterated DMSO, 0.06 mL of a relaxation agent solution chromium (III) acetylacetonate (0.016 M), and an internal standard (IS), trioxane (IS: lignin ratio was 1:10, *w*/*w*). The final solution was transferred into an NMR tube. A total of 16 samples were analysed by ^13^C-NMR. The 13C-NMR spectra were recorded at 25 °C in a Bruker Avance 600 MHz. Inverse gate detection and a 90° pulse width were used. A 1.1 s acquisition time and a 2.0 s relaxation delay were used, and 20,000 scans were collected. The spectra were Fourier transformed, phased, calibrated and the baseline was manually corrected by using a polynomial function [31].

For the quantification of the hydroxyl groups (phenolic and aliphatic) and carboxyl groups, ^31^P-NMR was used. The procedure for the sample preparation was the same as used in the literature [33]. For each ^31^P-NMR analysis, 40 mg of lignin was dissolved in 0.4 mL in a mixture of pyridine and deuterated chloroform (1.6:1, *v*/*v*). A total of 50 μL of chromium (III) acetylacetonate solution (11.4 mg/mL) and 100 μL of an IS solution (ca 0.12 M) were added. The internal standard to lignin was about 0.3 μmol/mg, in agreement with the standard protocols. Lastly, 100 μL of 2-chloro-4,4,5,5-tetramethyl-1,3,2-dioxaphospholane (TMDP) was added to the mixture, stirred for a few minutes, and transferred into an NMR tube, and submitted for NMR acquisition. For the 2D HSQC experiments, approximately 100 mg of each lignin sample was dissolved in 0.6 mL of deuterated DMSO. 

The spectra for each analysis were recorded In a Bruker Avance II+ 600 MHz spectrometer. For the ^31^P-NMR experiments, an acquisition time of 1.0 s was used and a 5.0 s relaxation delay. About 500 scans were collected. The spectra were phased and calibrated using the signal of the water-derivatized product at 132.2 ppm. The baseline was corrected [33]. For the 2D HSQC experiments, the HSQCETGPSISP.2 pulse sequence was used. The spectrometer was equipped with a 5 mm double resonance broadband BBI inverse probe, and a coupling constant J1 C-H of 147 Hz was used. The experimental parameters used were found in the literature [38].

**Gel Permeation Chromatography.** GPC analysis was performed by dissolving the dry lignin samples in DMSO/LiBr (0.5% *w*/*v*) after shaking overnight. Prior to GPC analysis, the solutions were filtered through 0.45 µm PTFE syringe filters. The equipment used and calculations are described in the literature [30].

**Elemental Analysis.** For the elemental analysis of CHNS, 1–2 mg of dry original lignin and lignin fractions were weighed into a tin capsule along with vanadium (V) oxide for the sulphur analysis. The equipment used was a Flash 2000 elemental analyser. 

**SEM.** The 8 lignin samples were freeze-dried and coated with a layer of gold/palladium, and analysed by scanning electron microscopy using an EOL JSM-6010 microscope at 10 kV. 

## 4. Conclusions

A fractionation method for Lignoboost Kraft lignin was developed using two different hydrotopic solutions with an initial concentration of 30 wt%. The fractionation method using SXS produced four different fractions with different molecular weights and yields. The fractionation using SCS produced three different fractions. The hydrotropic solutions can be recycled and reused several times, making this a non-toxic process to produce tailored lignin fractions. In future work, these fractions will be further analysed to verify if the Mw and different amounts of functional groups show different lignin reactivities. The possible applications for these lignin fractions range from binders/adhesives to cosmetics and biomedical applications. Research into the effect on the Mw of the interactions between the system water/hydrotrope/lignin could also be studied, creating a new research line to fractionate technical lignin with non-hazardous, reusable, and biodegradable hydrotropes into tailored Mw fractions. 

## Figures and Tables

**Table 1:** Yields of the different fractions obtained from the fractionation of Kraft lignin using SXS and SCS.

Hydrotrope	Fraction Name	% Fraction Yield
wt% SXS	16 wt%	72.2 ± 0.4
	14 wt%	10.8 ± 0.2
	12 wt%	2.5 ± 0.3
	10 wt%	1.1 ± 0.3
wt% SCS	10 wt%	5.1 ± 0.7
	8 wt%	78.9 ± 0.9
	6 wt%	5.2 ± 0.4

**Table 2:** GPC results for the fractions obtained from SXS and SCS fractionations.

Fraction Name	Mn (kDa)	Mw (kDa)	PDI (Mw/Mn)
Pure Lignin	2.3	24.5	10.4
16% SXS	3.2	24.6	7.6
14% SXS	2.6	20.6	7.9
12% SXS	3.0	9.7	3.2
10% SXS	2.4	7.3	3.0
10% SCS	2.8	24.0	8.4
8% SCS	4.2	21.3	5.0
6% SCS	4.1	19.0	4.6

**Table 3:** Average % of C, H, N and S in LKL and the lignin fractions.

Sample		$\bar{x}$	s (%)
LKL	C	64.10	0.17
	H	5.68	0.04
	N	0.24	0.02
	S	1.97	0
			
16 wt% SXS	C	63.94	0.04
	H	5.75	0.02
	N	0.25	0.01
	S	2.04	0.02
			
14 wt% SXS	C	65.73	0.06
	H	5.79	0.04
	N	0.26	0.01
	S	1.69	0.02
			
12 wt% SXS	C	65.19	0.11
	H	5.78	0.04
	N	0.25	0.01
	S	1.66	0
			
10 wt% SXS	C	65.63	0.15
	H	5.81	0.02
	N	0.26	0.01
	S	1.65	0.01

10 wt% SCS	C	64.80	0.06
	H	5.82	0.01
	N	0.24	0.01
	S	2.24	0.02

8 wt% SCS	C	65.28	0.13
	H	5.87	0
	N	0.25	0.02
	S	2.15	0.04

6 wt% SCS	C	65.34	0.12
	H	5.85	0.01
	N	0.24	0.01
	S	1.79	0.01

**Table 4:** Quantification of lignin interunit linkages by ^13^C NMR in acetylated lignin samples.

Per 100 Ar Units	β-O-4′	β-5	β-β
pure lignin	11.4	3.1	3.4
16 wt% SXS	12.2	3.3	3.9
14 wt% SXS	12	3.4	3.9
12 wt% SXS	9.8	2.8	3.3
10 wt% SXS	6.6	1.8	2.4
10 wt% SCS	10.1	2.7	3.5
8 wt% SCS	10.2	3	3.7
6 wt% SCS	10.2	2.9	3.6

## Data Availability

Not applicable.

## Supplementary Materials

The following supporting information can be downloaded at: https://www.mdpi.com/article/10.3390/molecules28020687/s1, Figure S1: ^13^C NMR spectra of original Lignoboost Kraft lignin non-acetylated (top) and acetylated (bottom); Figure S2. ^13^C NMR spectra of non-acetylated original LKL and fractions obtained from SCS and SXS fractionation; Figure S3. ^13^C NMR spectra of acetylated original LKL and fractions obtained from SXS fractionation; Figure S4. ^13^C NMR spectra of acetylated original LKL and fractions obtained from SCS fractionation; Figure S5. ^31^P NMR of original lignin and SXS fractions; Figure S6. ^31^P NMR of original lignin and SCS fractions; Figures S7–S13. 2D HSQC spectra of LKL and its fractions; Figure S14. ^13^C NMR spectra of pure SCS and SXS; Figure S15. ^1^H NMR spectra of pure SCS and SXS.
