# Supplementary material for: Advanced Fractionation of Kraft Lignin by Aqueous Hydrotropic Solutions
Source: Molecules. 2023 Jan 10;28(2):687. doi: 10.3390/molecules28020687 (PMC9867506; doi:10.3390/molecules28020687)
Supplement: Supplementary file 1 [file molecules-28-00687-s001.zip › molecules-2128066-supplementary.pdf]

Supplementary information

# Advanced Fractionation of Kraft Lignin by Aqueous Hydrotropic Solutions

**Rita Gaspar [1], Marcelo Coelho dos Santos Muguet [2] and Pedro Fardim [1,*]**

**<u>$^{13}$C NMR spectra of non-acetylated lignin samples</u>**

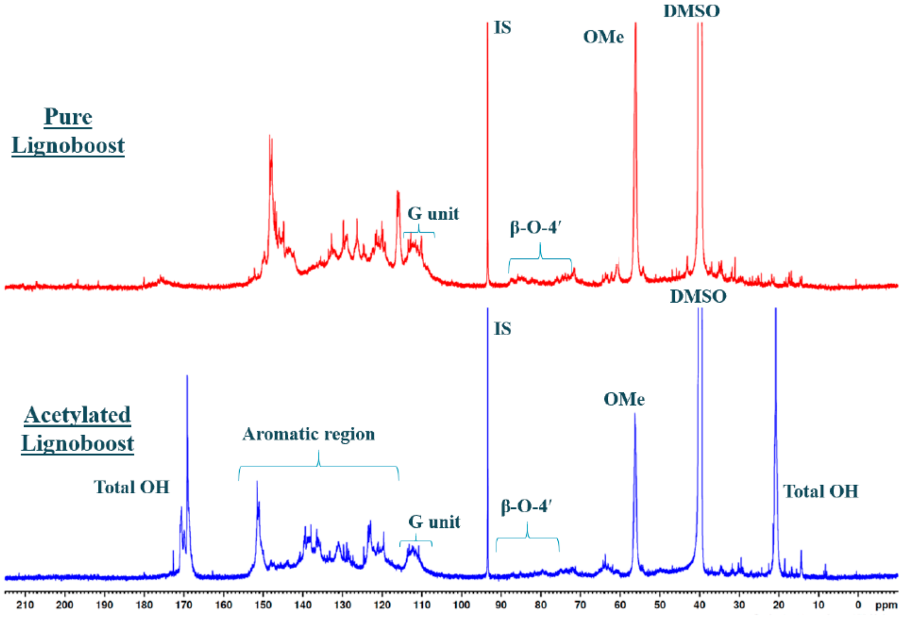


*Figure S1. $^{13}$C NMR spectra of original Lignoboost Kraft lignin non-acetylated (top) and acetylated (bottom).*

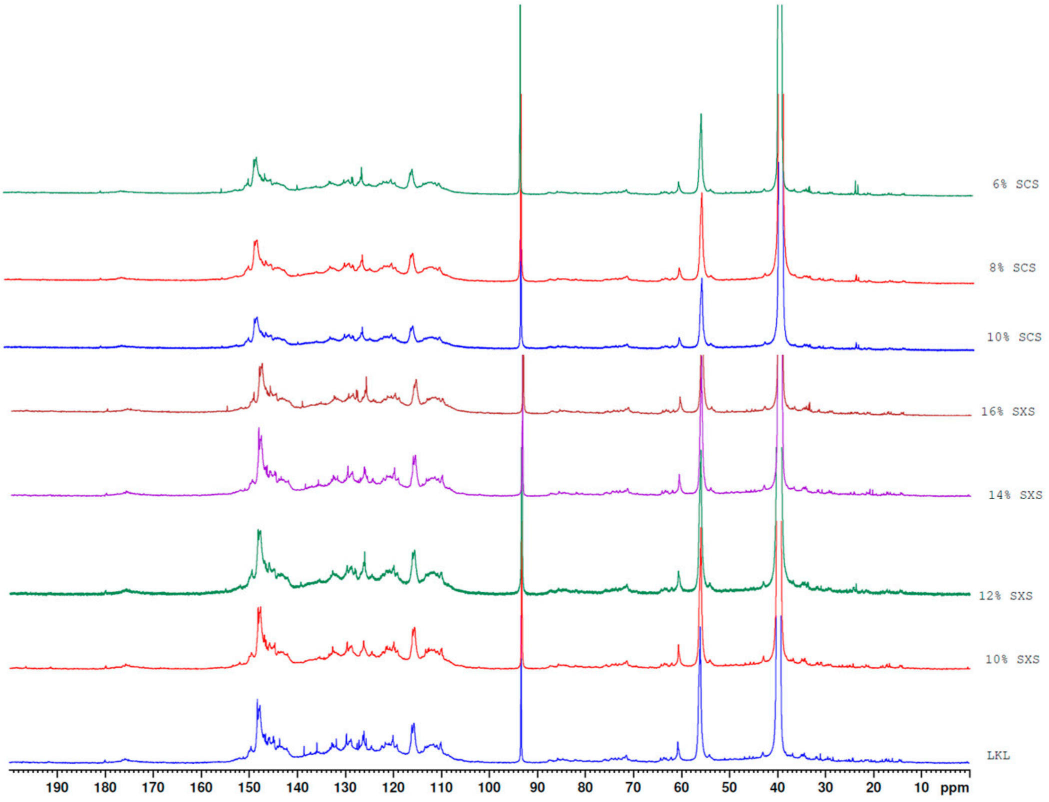


*Figure S1.* $^{13}$*C NMR spectra of non-acetylated original LKL and fractions obtained from SCS and SXS fractionation.*

## $^{13}$C NMR spectra of acetylated lignin samples

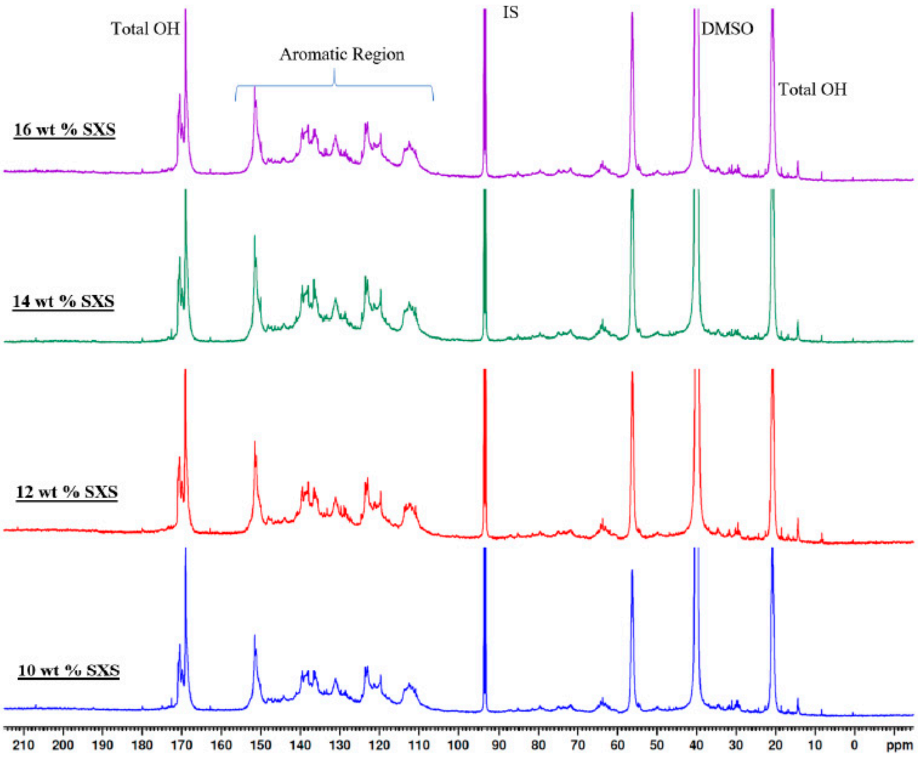


*Figure S2.* $^{13}$*C NMR spectra of acetylated original LKL and fractions obtained from SXS fractionation.*

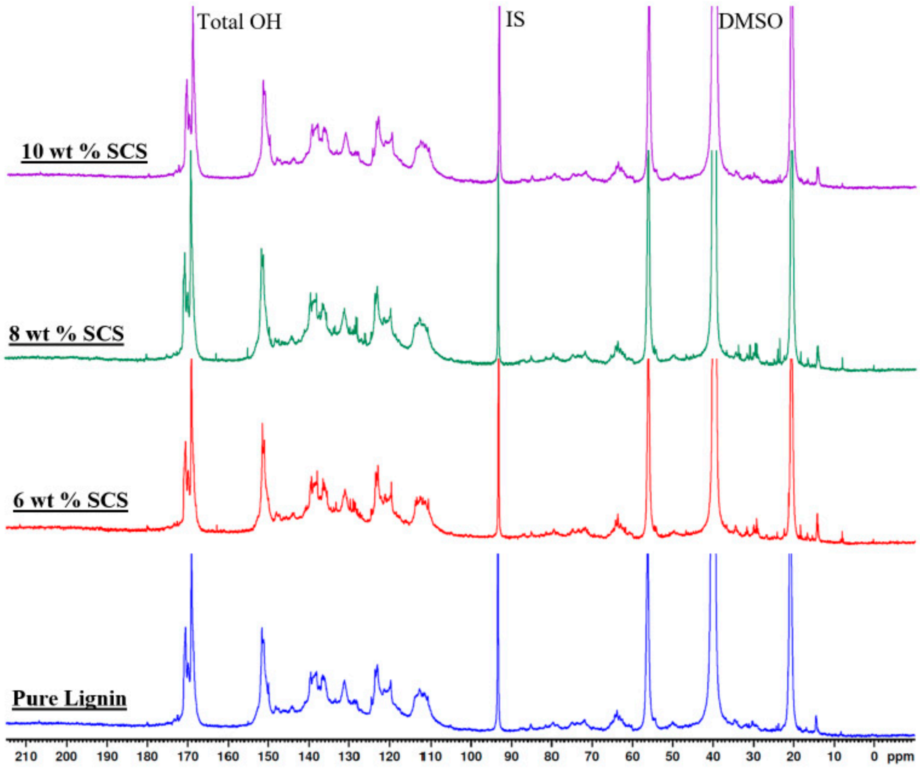


*Figure S3. $^{13}C$ NMR spectra of acetylated original LKL and fractions obtained from SCS fractionation.*

**$^{31}P$ NMR spectra of lignin samples**

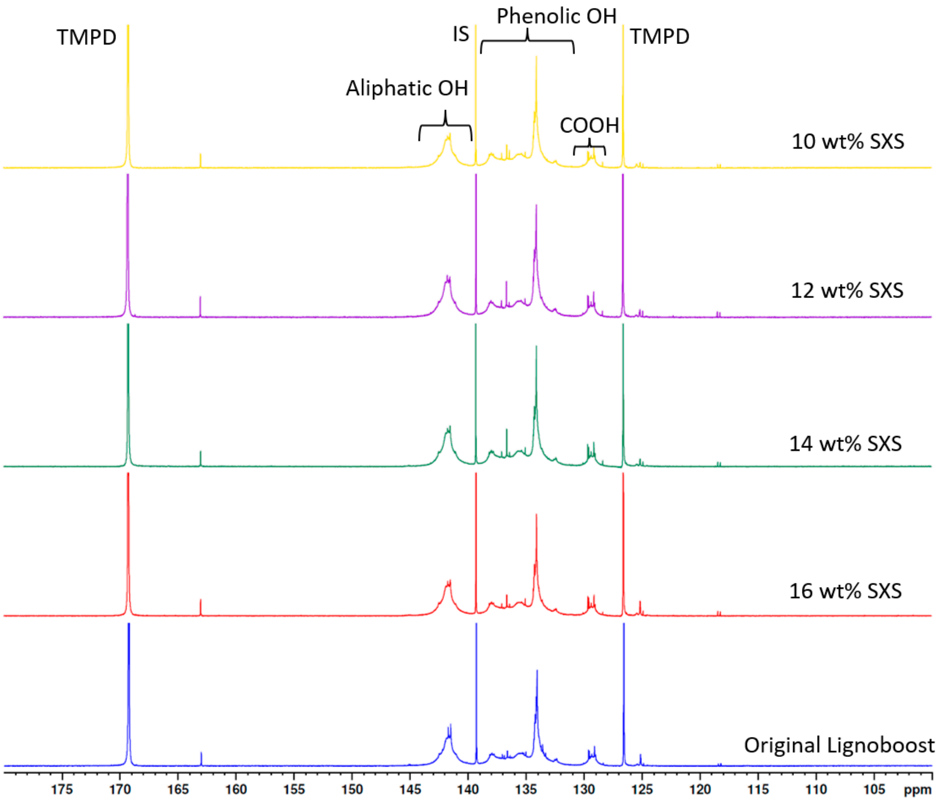


*Figure S4. $^{31}P$ NMR of original lignin and SXS fractions*

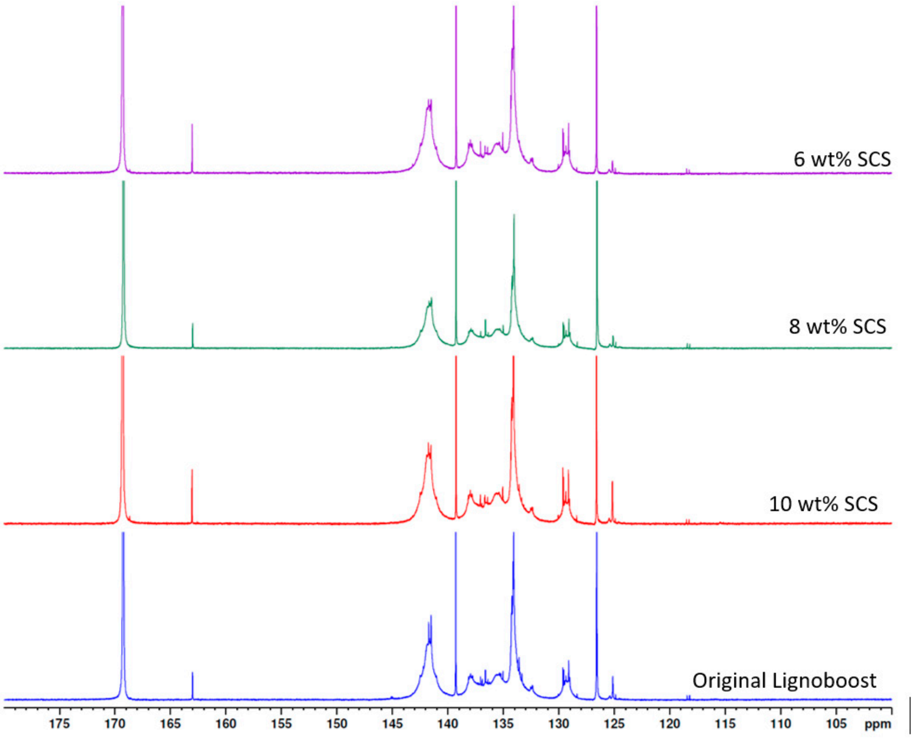


*Figure S5. $^{31}P$ NMR of original lignin and SCS fractions.*

## ***2D HSQC spectra of lignin samples***

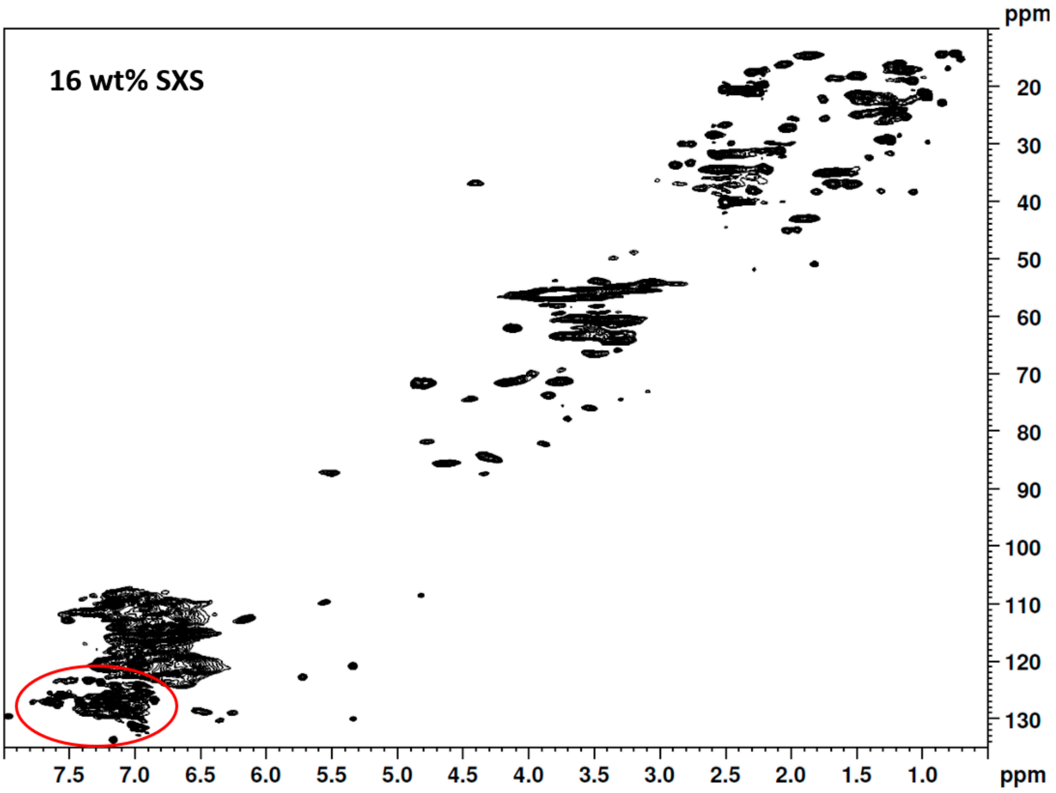


*Figure S6. 2D HSQC spectrum of 16 wt% SXS fraction.*

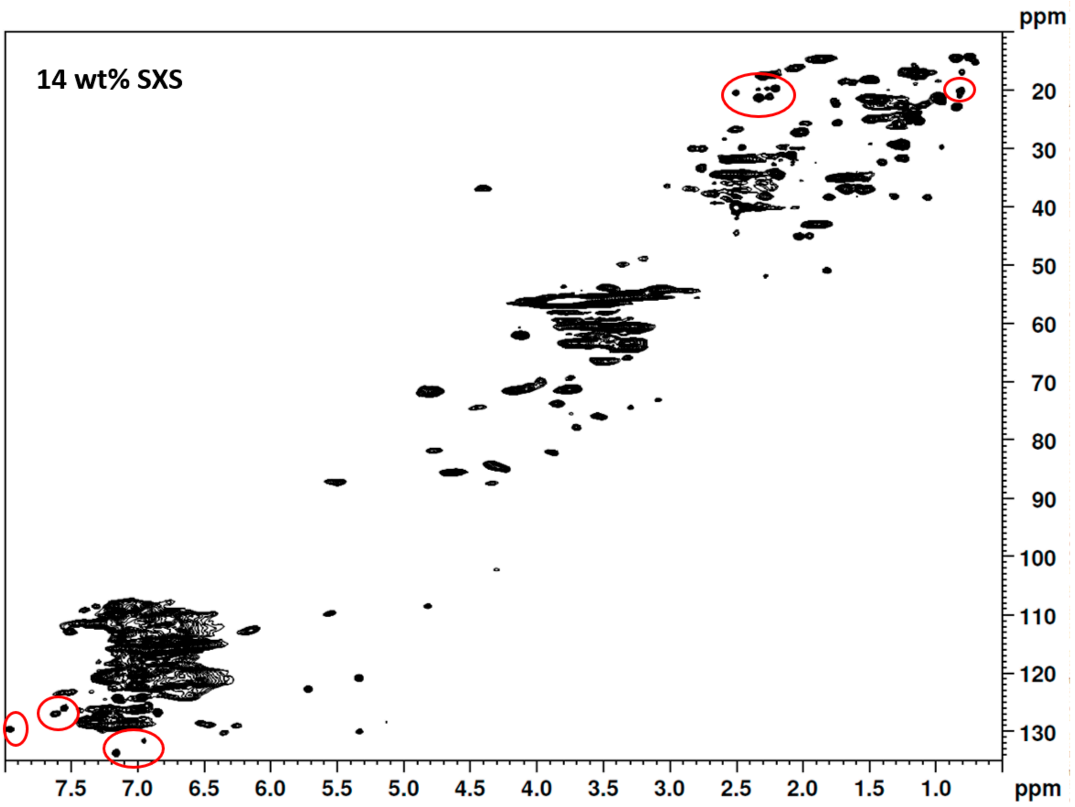


*Figure S7. 2D HSQC spectrum of 14 wt% SXS fraction.*

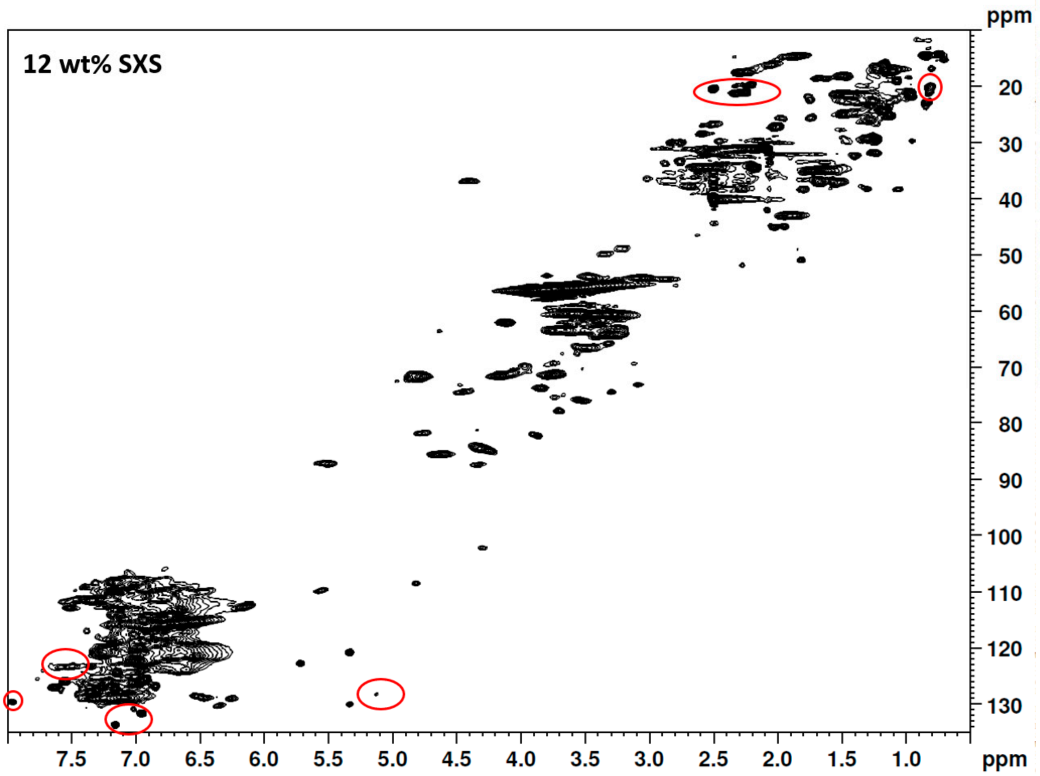


*Figure S8. 2D HSQC spectrum of 12 wt% SXS fraction.*

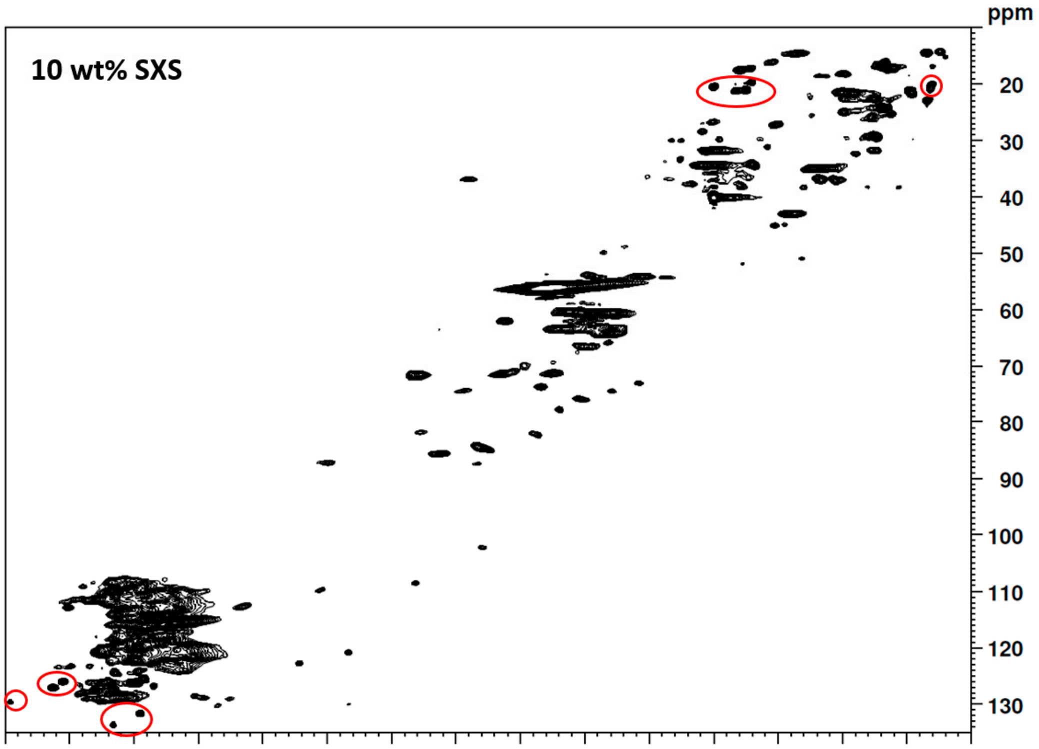


*Figure S9. 2D HSQC spectrum of 10 wt% SXS fraction.*

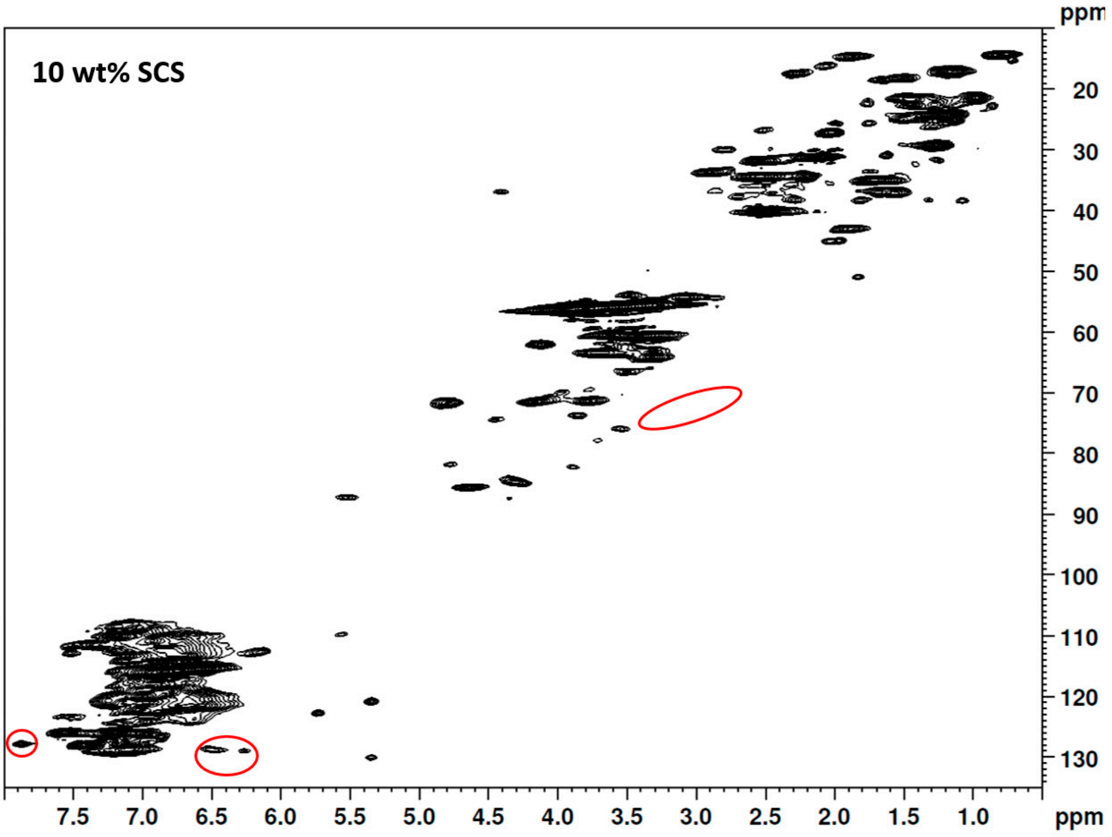


*Figure S10. 2D HSQC spectrum of 10 wt% SCS fraction.*

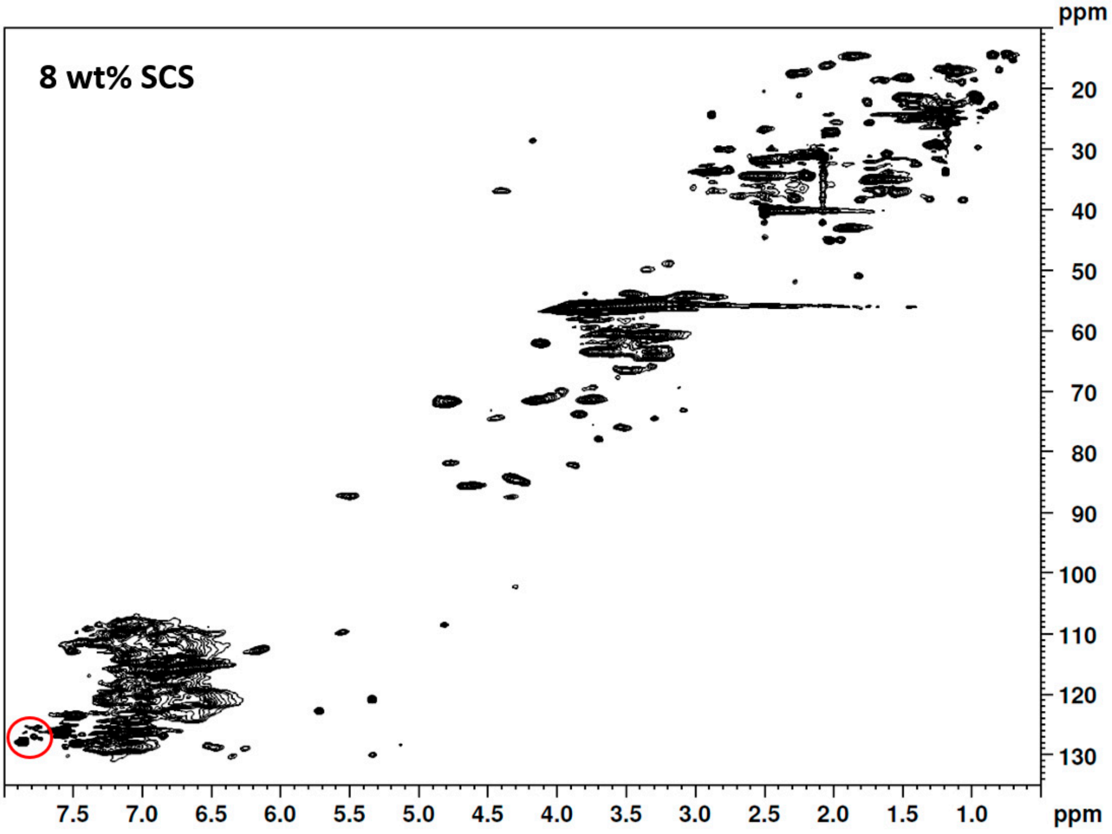


*Figure S11. 2D HSQC spectrum of 8 wt% SCS fraction.*

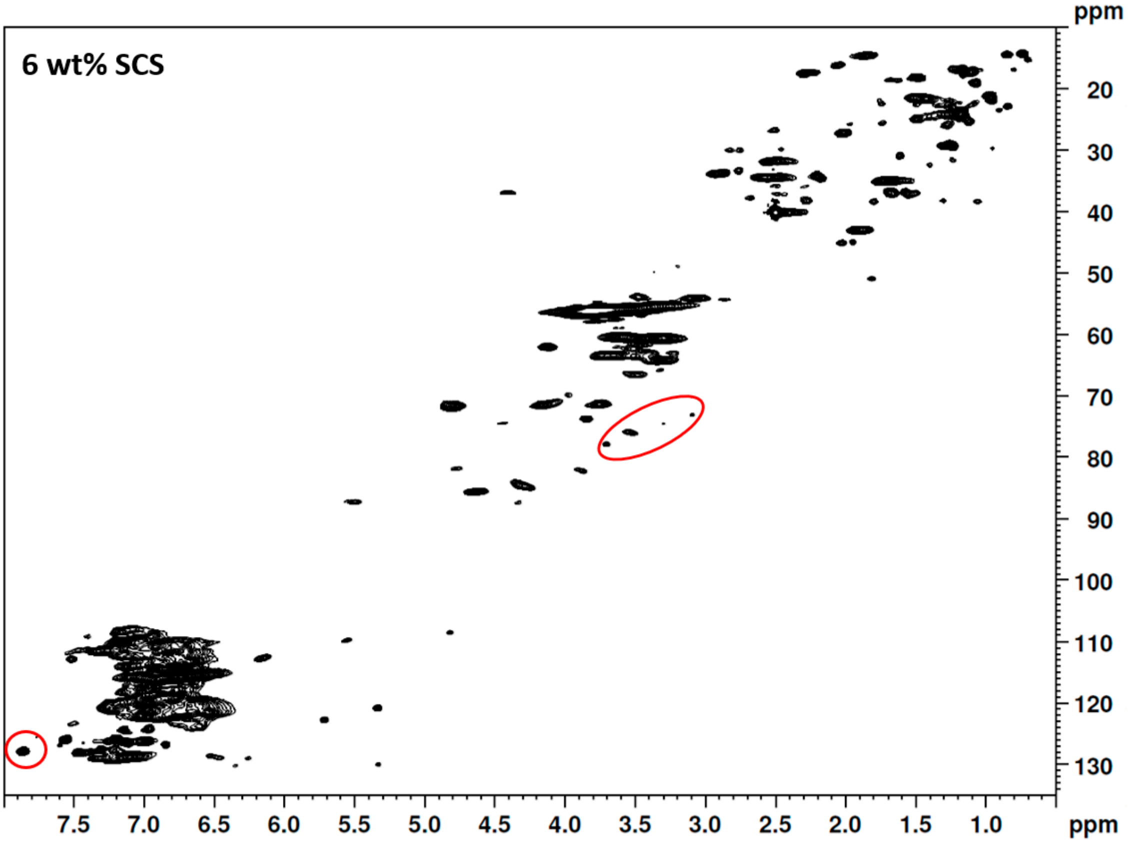


*Figure S12. 2D HSQC spectrum of 6 wt% SCS fraction.*

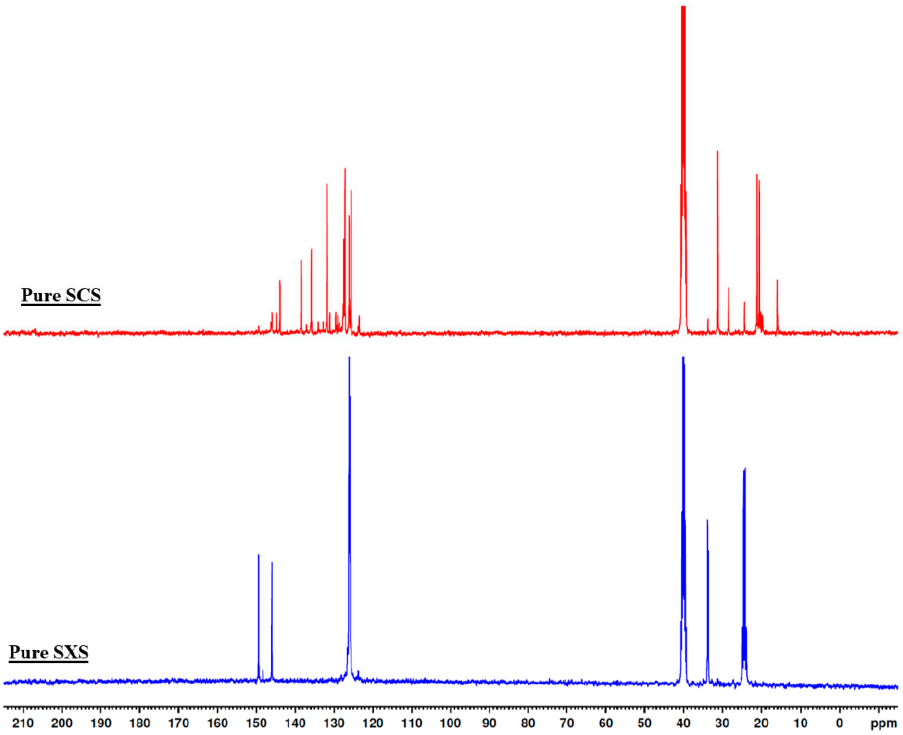


*Figure S13. $^{13}$C NMR spectra of pure SCS and SXS.*

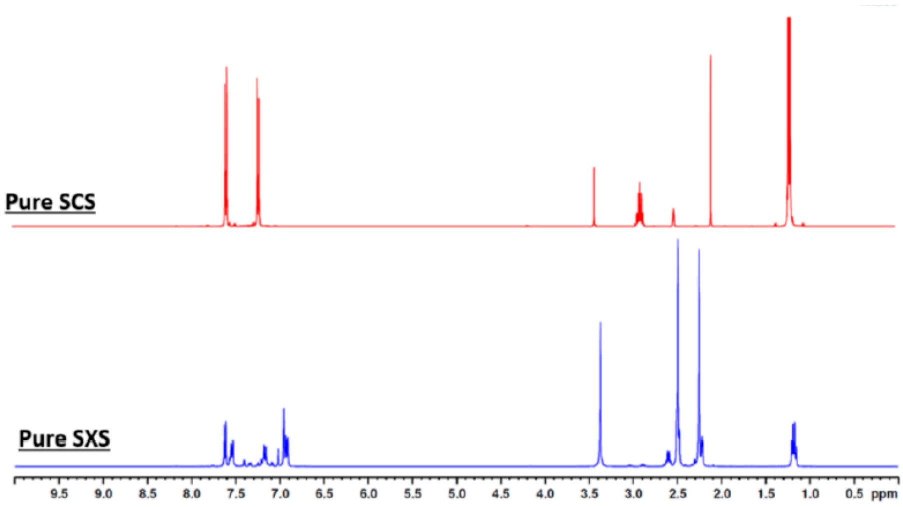


*Figure S14. $^{1}$H NMR spectra of pure SCS and SXS.*
